# Beneficial Soil Bacterium *Pseudomonas frederiksbergensis* OS261 Augments Salt Tolerance and Promotes Red Pepper Plant Growth

**DOI:** 10.3389/fpls.2017.00705

**Published:** 2017-05-04

**Authors:** Poulami Chatterjee, Sandipan Samaddar, Rangasamy Anandham, Yeongyeong Kang, Kiyoon Kim, Gopal Selvakumar, Tongmin Sa

**Affiliations:** ^1^Department of Environmental and Biological Chemistry, Chungbuk National UniversityCheongju, South Korea; ^2^Department of Agricultural Microbiology, Agricultural College and Research Institute, Tamil Nadu Agricultural UniversityMadurai, India; ^3^Horticultural and Herbal Crop Environment Division, National Institute of Horticultural and Herbal Science, Rural Development AdministrationWanju, South Korea

**Keywords:** plant growth promotion, salt stress, *Pseudomonas frederiksbergensis*, antioxidant enzyme, bio-fertilizer

## Abstract

Soil salinity, being a part of natural ecosystems, is an increasing problem in agricultural soils throughout the world. *Pseudomonas frederiksbergensis* OS261 has already been proved to be an effective bio-inoculant for enhancing cold stress tolerance in plants, however, its effect on salt stress tolerance is unknown. The main aim of the present study was to elucidate *P. frederiksbergensis* OS261 mediated salt stress tolerance in red pepper. The plants were exposed to a salt stress using NaCl at the concentrations of 50, 100, and 150 mM after 12 days of transplantation, while plant growth and enzyme activity were estimated 50 days after sowing. The height in *P*. *frederiksbergensis* OS261 inoculated plants was significantly increased by 19.05, 34.35, 57.25, and 61.07% compared to un-inoculated controls at 0, 50, 100, and 150 mM of NaCl concentrations, respectively, under greenhouse conditions. The dry biomass of the plants increased by 31.97, 37.47, 62.67, and 67.84% under 0, 50, 100, and 150 mM of NaCl concentrations, respectively. A high emission of ethylene was observed in un-inoculated red pepper plants under salinity stress. *P*. *frederiksbergensis* OS261 inoculation significantly reduced ethylene emission by 20.03, 18.01, and 20.07% at 50, 100, and 150 mM of NaCl concentrations, respectively. Furthermore, the activity of antioxidant enzymes (ascorbate peroxidase, superoxide dismutase, and catalase) also varied in the inoculated red pepper plants. Salt stress resistance in the bacterized plants was evident from the improved antioxidant activity in leaf tissues and the decreased hydrogen ion concentration. Thus, we conclude that *P*. *frederiksbergensis* OS261 possesses stress mitigating property which can enhance plant growth under high soil salinity by reducing the emission of ethylene and regulating antioxidant enzymes.

## Introduction

Abiotic stresses exert a serious impact on crop productivity throughout the world. Soil salinity is an alarming stress, which limits plant growth and affects crop production to a large extent (Allakhverdiev et al., 2000; Bano and Fatima, 2009; Bacilio et al., 2016). The high amount of salt in soil disturbs the ionic balance in plant cells and decreases water uptake capacity in plants (Cheng et al., 2007; Singh and Jha, 2016; Stavridou et al., 2016; Yaish et al., 2016). In principle, high amounts of potassium or calcium ions are needed for the proper functioning of plant metabolism, whereas increased uptake of sodium ion due to high salinity reduces the ionic uptake of potassium and calcium ions (Cheng et al., 2007), thereby negatively affecting the growth of plants. It disturbs plant cell division and elongation and reduces the rate of photosynthesis (Munns and Tester, 2008; Forieri et al., 2016). This finally induces the generation of reactive oxygen species (ROS). The generated ROS negatively affect plant growth and development (Bojórquez-Quintal et al., 2014). Thus developing salt stress tolerant plants has been a highly desirable goal but there has been a little success to date; as only a few major genetic determinants of salt stress have been identified (Munns and Tester, 2008; Schubert et al., 2009). The alternate strategy is to introduce salt-tolerant microbes into plants under saline conditions. Beneficial microbes, such as plant growth promoting rhizobacteria (PGPR), which live in and around the root zone (rhizosphere), have been investigated as the means to overcome salinity stress. Most of the bacterial genera, such as *Agrobacterium, Azospirillum, Bacillus*, and *Rhizobium*, have already been reported to increase salt tolerance in plants (Hamdia et al., 2004; Ahmad et al., 2012; Bharti et al., 2013). They are known to stimulate plant growth through a variety of mechanisms such as the production of phytohormones, ACC deaminase (Siddikee et al., 2015), exopolysaccharide (EPS) (Siddikee et al., 2011), fixation of atmospheric nitrogen (Islam et al., 2013), and by solubilizing phosphate (Tank and Saraf, 2010). The stress usually induces ethylene emission (Morgan and Drew, 1997) and PGPR play a major role in reducing ethylene in plants via the action of ACC deaminase that cleaves ACC to α-ketobutyrate and ammonia (Glick et al., 1998). An abiotic stress induces ROS as a result of the stress signal within the plant. Mostly, H_2_O_2_, O^2-^, and OH^-^ are generated, which damage biological macromolecules: DNA, RNA, and proteins. ROS not only affect plants at a molecular level but also hamper main physiological activities. There are some antioxidant enzymes that can control the production of ROS under stressful conditions (Munne-Bosch and Pinto-Marijuan, 2016). Superoxide dismutase (SOD), ascorbate peroxidase (APX), and catalase (CAT) are the main enzymes that play a major role in the self-defense in plants (Chojak-Koźniewska et al., 2017). SOD primarily converts superoxide radicals into hydrogen peroxide, thus defending the plants from oxidative damage. APX mainly works in peroxisomes where it helps in the breakdown of H_2_O_2_. CAT lowers the hydrogen peroxide level by converting it into water and oxygen (Habib et al., 2016). The microbes provide plants resistance to stress by enhancing the activity of the antioxidant enzymes and other non-enzymatic antioxidants (Gururani et al., 2013).

Pepper (*Capsicum annuum* L. family Solanaceae) is widely grown in the different parts of the world and is one of the most popular vegetables in South Korea. It is an important ingredient of many traditional Korean foods, including Kimchi (Sang et al., 2008; Patra et al., 2016). Pepper is being cultivated in greenhouses as well as in fields since a long time. The repeated cultivation of pepper can increase soil salinity through the accumulation of organic fertilizers and pesticides, and as a result, an agricultural land can become highly saline, unsuitable for agriculture. High soil salinity causes major economic losses by reducing plant yield. In South Korea, a low temperature which is unfavorable for plant growth dominates 6 months of the year making productive agriculture possible only in summer. In addition to that, the crop production in the coastal regions of South Korea is also limited due to high salinity. This leads to an economically unfavorable situation. Recently, various approaches have been suggested to control salinity stress, one of them is the use of beneficial PGPR, which can enhance plant growth and productivity by eliciting salt-stress tolerance to host plants (Yang et al., 2009; Siddikee et al., 2010; Dodd and Pérez-Alfocea, 2012). In order to maximize the crop yield and make it economically favorable, it would be convenient to use a bacterial strain capable of mitigating both low temperature and salinity stress. *P. frederiksbergensis* OS261, a PGPR, and member of the group Gammaproteobacteria, has been proved to increase the fitness of tomato plants under cold stress conditions (Subramanian et al., 2016) and also displayed its ability to colonize in plant parts of red pepper (Sandipan et al., 2016) and tomato (Subramanian et al., 2015). Even though *P. frederiksbergensis* OS261 has been proved to be an effective bio-inoculant for enhancing cold stress tolerance in plants (Subramanian et al., 2016), its effect on increasing salt stress tolerance still remains largely unknown. This work was aimed to evaluate *P. frederiksbergensis* OS261 as an effective inoculant for enhancing salt stress tolerance in plants through the production of ACC deaminase, reduction of stress ethylene level, and induction of antioxidant enzymes.

## Materials and Methods

### Bacterial Strain and Culture Conditions

*Pseudomonas frederiksbergensis* OS261 (GenBank Accession no. KF424313, 16S ribosomal RNA gene) was previously isolated from the experimental fields of Chungbuk Agricultural Research and Extension Services, Ochang-eup, South Korea (36°43′ N; 127°27′E). This strain was selected on the basis of plant growth promoting effects and ACC deaminase activity (Subramanian et al., 2016). For inoculum preparation, a single colony of *P*. *frederiksbergensis* OS261 grown on a nutrient agar (NA) plate was transferred to 5 mL nutrient broth (NB) and incubated at 30°C on a shaker (150 rpm) for 24 h. After the incubation, 1.0 mL of the culture (∼1.0 × 10^8^ CFU mL^-1^) was transferred to 50 mL fresh NB and allowed to grow for further 24 h. The culture was centrifuged at 5000 rpm for 5 min at 4°C, and the pellet was washed twice with 30 mM MgSO_4_. The pellet was resuspended in 30 mM MgSO_4_ to an optical density of 0.8 at 600 nm (cell count ∼1.0 × 10^8^ CFU mL^-1^).

### Plant Experiments

#### Seed Sterilization and Bacterization

Red pepper (*C. annuum* L. cv. Bulmat) seeds (Syngenta seeds, Seoul, Republic of Korea) were surface sterilized with 70% ethanol for 30 s and 2% sodium hypochlorite for 1 min, followed by rinsing for several times with sterile deionized water. The bacterial cultures were grown in NB media at 30°C for 24 h with 150 rpm. The grown culture was harvested by centrifuging at 5000 rpm for 5 min at 4°C and the cell pellets were resuspended in 30 mM MgSO_4_ to obtain an optical density of 0.8 at 600 nm (cell count ∼1.0 × 10^8^ CFU mL^-1^). The bacterization was performed by soaking the seeds in bacterial suspension for 4 h whereas the control seeds were soaked only in MgSO_4_.

#### Gnotobiotic Pouch Experiments

The red pepper seeds were surface sterilized and bacterized as mentioned above. After decanting the suspension, the seeds were transferred to growth pouches (six seeds per pouch), with six replications (i.e., six pouches) per treatment. The pouches were then kept in a growth chamber (DS 54 GLP, DASOL Scientific Co., Ltd., Korea) at 25°C with 12 h day-night photoperiod for 7 days (relative humidity 70% and light intensity 18 μmol m^-2^ s^-1^). After 7 days of plant growth, 0, 50, 100, and 150 mM of sodium chloride solutions prepared in 1X Hoagland solution were used to induce salt stress. The effect of inoculation on early growth promotion was determined by measuring the root and shoot length after 14 days of sowing (DAS). Total dry matter accumulation was measured after drying the seedlings in an oven at 70°C for 48 h.

#### Determination of Ethylene Emission

The ethylene emission from the red pepper seedlings was measured following the protocol of Mayak et al. (2004) with some modifications. The seed bacterization was performed as mentioned above. Following the bacterization, 30 seeds were imbibed in either a bacterial suspension or 30 mM MgSO_4_ for 2 h, the suspension or buffer was drained, the seeds were placed inside 120 mL narrow neck bottles on a piece of filter paper and 2 mL of de-ionized water was added to each bottle. After 8 days, the excess liquid was drained and 2 mL of respective salt solution (50, 100, and 150 mM) was added. The seeds those were imbibed in buffer and were not treated with salt served as un-inoculated control. After 4 h of salt addition, the narrow neck bottles were closed for 2 h with a rubber septum and 1 mL sample from the headspace was injected into a gas chromatograph (dsCHROM 6200, Donam Instruments Inc., Republic of Korea) packed with a Porapak-Q column and equipped with a flame ionization detector. The oven, injection, and detection temperatures were set at 40, 150, and 250°C, respectively. Six replicates were used for each treatment.

#### Greenhouse Experiments

The bacterized and non-bacterized red pepper seeds were sown in seedling trays containing 40 g of nursery soil (Nongwoo-Bio Co., Ltd., Yeoju-gun, Gyeonggi-do, Republic of Korea; composition: 58.8% coco peat, 17% peat moss, 10% perlite, 10% vermiculate, 4% zeolite, 0.004% pyroligneous acid and 0.01% wetting agent) and incubated in the greenhouse at a temperature range of 25–30°C with 50–70% relative humidity and a 15–9 h day/night period. Seven days old red pepper seedlings were transplanted into plastic pots (16 cm × 16 cm) containing 500 g of nursery soil. After transplanting, the plants were allowed to grow under normal conditions in the greenhouse. The bacterial inoculum was prepared as mentioned above and the inoculation was carried out in the soil at 14 DAS by adding 10 mL of the bacterial suspension (cell count ∼1.0 × 10^8^ CFU mL^-1^) near the root zone. Five days after applying the bacterial suspension, salt stress was induced gradually by applying 25 mM of sodium chloride solution to each pot on alternative days to avoid osmotic shock; and the desired salt concentrations of 50, 100, and 150 mM were achieved after 2, 6, and 10 days, respectively. Parallel controls were maintained by irrigating with tap water. The leaching of water from the pots was prevented by retaining the soil water to a level below water holding capacity. The soil electrical conductivity of 0, 50, 100, and 150 mM treatments were 1.31 ± 0.04, 6.23 ± 0.07, 10.23 ± 0.35, and 16.23 ± 0.21 dS/m, respectively, at the time of harvest. Fifty days after the sowing, the plants were uprooted and the length of the plants was measured. Additionally, the number of leaves per plant was also recorded. The total dry biomass of the plants was determined after drying the plants in an oven at 70°C for 72 h.

#### Determination of Antioxidant Enzymatic Activity

Antioxidant enzymes were measured from a crude enzyme extract derived from red pepper leaves (approximately ∼1 g) obtained from the pot culture plants that were harvested at 50 DAS. Briefly, the fresh leaves were ground using liquid nitrogen and the ground leaf samples were stored at –80°C. The ground leaf samples (0.5 g) were homogenized in ice using 10 mL of 50 mM of potassium phosphate buffer, 1% (w/v) polyvinylpyrrolidone (pH 7.8) then incubated for 10 min at 4°C. The homogenate was filtered using Advantech Qualitative Filter Papers (110 mm) and centrifuged (VS–24SMTi, High Speed Refrigerated Centrifuge, Vision Scientific Co. Ltd., Korea) at 4000 × *g* for 15 min at 4°C. The supernatant was considered as the enzyme extract and was used for the determination of enzyme activities. The CAT activity (EC 1.11.1.6) was determined by a hydrogen peroxide assay based on the formation of its stable complex with ammonium molybdate (Goth, 1991). Briefly, 0.2 mL of the enzyme extract was incubated in 1 mL reaction mixture containing 65 mM hydrogen peroxide in 60 mM sodium phosphate buffer at room temperature for 4 min. The reaction was terminated using 1 mL of 32.4 mM of ammonium molybdate and the concentration of the yellow complex formed during the reaction was measured at 405 nm. SOD (EC 1.15.1.1) and APX (EC 1.11.1.11) activities were measured according to Ding et al. (2011). In the case of SOD, the assay mixture contained 50 mM phosphate buffer (pH 7.8), 9.9 mM L-methionine, 57 μM NBT, 0.025% (w/v) Triton X-100, 0.0044% (w/v) riboflavin and the enzyme extract. The SOD activity was measured at 560nm by following the decrease in the absorbance due to the photochemical reduction of nitro-blue tetrazolium (NBT). The APX activity was evaluated by the oxidative conversion of ascorbic acid to dehydroascorbate with regard to the decrease in the absorbance. It was calculated by the means of extinction coefficient 2.8 mM^-1^ cm^-1^ at 290 nm. The reaction mixture contained 50 mM potassium phosphate buffer (pH 7.0), 0.3 mM ascorbic acid, 0.1 mM H_2_O_2_, 0.1 mM EDTA and 50 μL of the enzyme extract. Hydrogen peroxide content in the leaves was evaluated according to the protocol of Theocharis et al. (2012).

#### Statistical Analysis

In this study, a completely randomized block design was employed for the early growth and greenhouse experiments. The data from experimental results were subjected to a two-way analysis of variance (ANOVA). The percentage data and the significant differences between the means were determined by Duncan’s Multiple Range Test (DMRT) at *P* < 0.05 using SAS package, Version 9.4.

## Results

### Effect of *P. frederiksbergensis* OS261 on the Growth of Red Pepper Seedlings under Gnotobiotic Conditions

**FIGURE 1:**
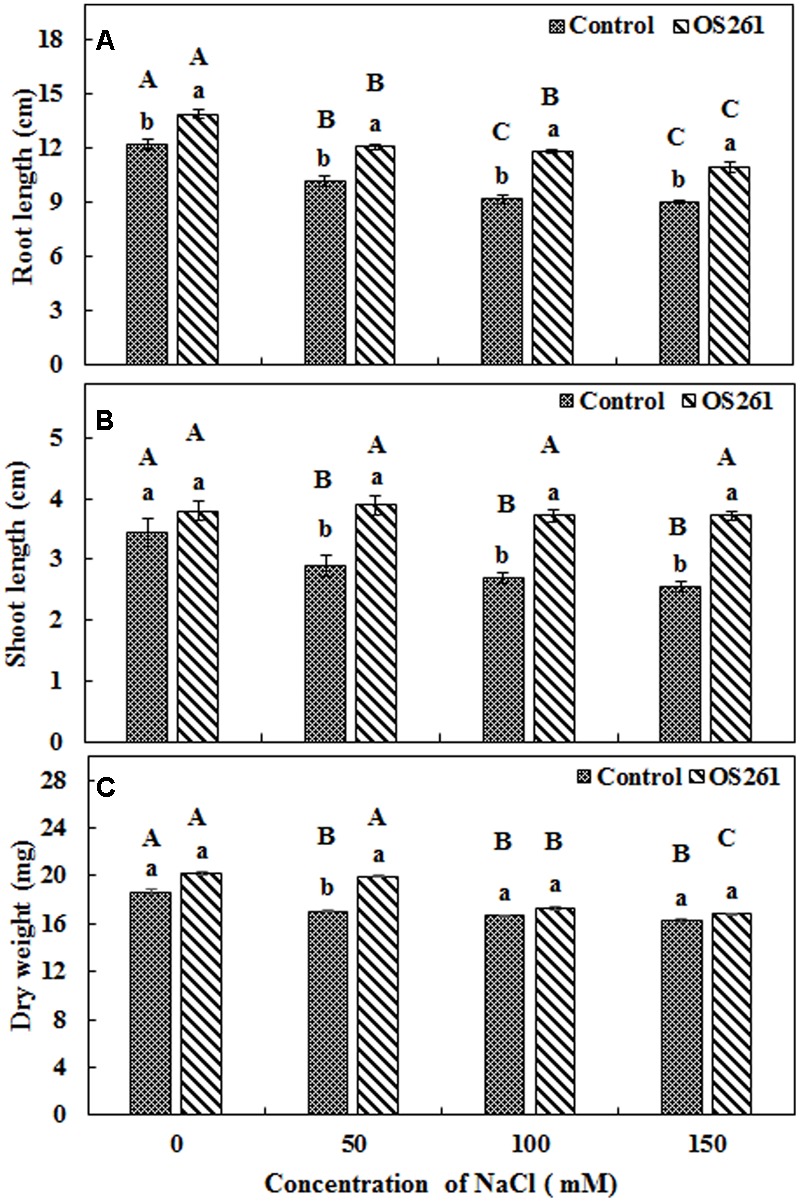
**Effect of inoculation of *Pseudomonas frederiksbergensis* OS261 on red pepper seedlings under gnotobiotic conditions. (A)** Root length, **(B)** Shoot length, **(C)** Dry weight. Different letters indicate significant differences *P* < 0.05 among the treatments at each salt levels (a, b) or among salt levels for each treatment: Control (A, B, C, D), OS261 (A, B, C, D). Each value represents the mean of six replicates ± standard error (SE).

The effects of inoculation of *P. frederiksbergensis* OS261 on growth of red pepper under gnotobiotic conditions are shown in **Figure 1**. The plants inoculated with the bacterium developed longer roots (**Figure 1A**) compared to the un-inoculated controls after a period of 14 days under salinity stress. The inoculation increased the root length by 13.93, 18.62, 28.26, and 22.22% under 0, 50, 100, and 150 mM of NaCl treatment, respectively, compared to the controls. On the other hand, the changes in shoot length (**Figure 1B**) were also noticeable. The bacterial inoculation increased the shoot length by 11.76, 34.48, 37.03, and 45.09% under 0, 50, 100, and 150 mM of salt treatment, respectively, compared to the controls. Similarly, the inoculated red pepper plants accumulated higher plant biomass (**Figure 1C**) compared to the un-inoculated controls under salt-stressed conditions with a significant increase in the salt stress induced by 50 mM NaCl.

### Determination of Ethylene Emission from Red Pepper Seedlings with and without Inoculation

**FIGURE 2:**
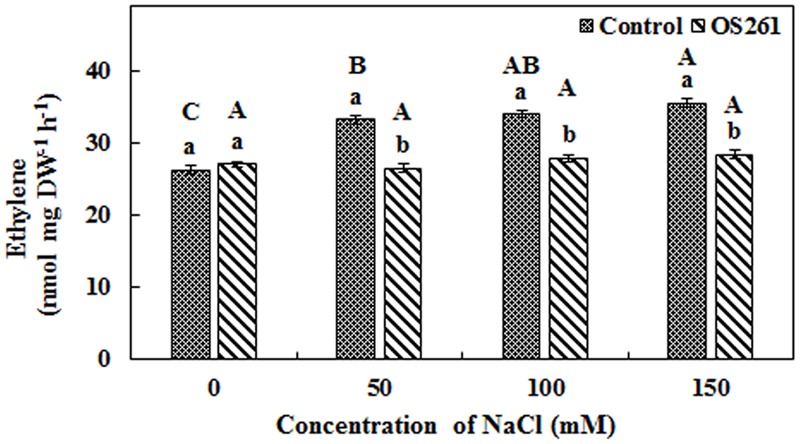
**Effect of *P. frederiksbergensis* OS261 on ethylene production by red pepper seedlings under 0, 50, 100, and 150 mM salinity stress.** Different letters indicate significant differences *P* < 0.05 among the treatments at each salt levels (a, b) or among salt levels for each treatment: Control (A, B, C, D), OS261 (A, B, C, D). Each value represents the mean of six replicates ± standard error (SE).

A higher amount of ethylene emission was observed from un-inoculated red pepper seedlings under 50, 100, and 150 mM of salt stress (**Figure 2**), but inoculation of *P. frederiksbergensis* OS261 reduced ethylene emission. It was observed that ethylene production increased by 20, 18, and 20.07% in un-inoculated red pepper seedlings at 50, 100, and 150 mM NaCl, respectively, compared to the inoculated red pepper seedlings.

### Effects of Inoculation with *P. frederiksbergensis* OS261 on the Growth of Red Pepper Plants under Salt Stress Conditions in the Greenhouse

**FIGURE 3:**
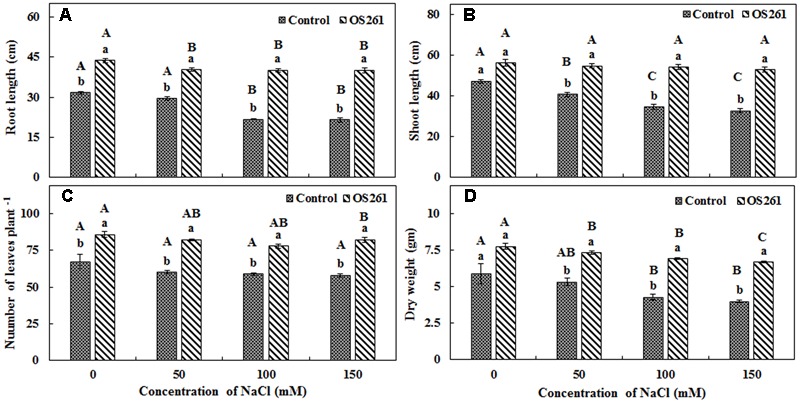
**Effect of inoculation of *P. frederiksbergensis* OS261 on red pepper plants under greenhouse conditions. (A)** Root length, **(B)** Shoot length, **(C)** Number of leaves per plant, **(D)** Dry weight. Different letters indicate significant differences *P* < 0.05 among the treatments at each salt levels (a, b) or among salt levels for each treatment: Control (A, B, C, D), OS261 (A, B, C, D). Each value represents the mean of three replicates ± standard error (SE).

In order to validate the results obtained in gnotobiotic conditions, similar experiments were performed under greenhouse conditions and the effect of inoculation on plant growth was checked by the means of plant height, dry biomass, and the number of leaves (**Figure 3**). The inoculated plants showed 37.79% increase in root length, 19.05% increase in shoot length and 31.97% increase in dry weight under normal conditions. Among the different levels of salt stress (50, 100, and 150 mM), the trends were similar, where inoculation increased root length (**Figure 3A**) by 36.44, 84.44, and 86.63%, respectively, as well shoot length (**Figure 3B**) by 34.35, 57.25, and 61.07%, respectively, compared to the un-inoculated controls. The number of leaves in each plant (**Figure 3C**) also increased significantly, contributing to the increase in dry biomass (**Figure 3D**), by 37.47, 62.67, and 67.84% under 50, 100, 150 mM of NaCl treatments in comparison to the un-inoculated plants. Overall, the inoculation resulted in a significant improvement in plant growth under both normal and salt-stressed conditions.

### Antioxidant Enzyme Activities and Hydrogen Peroxide Content in Red Pepper Plants Inoculated with *P. frederiksbergensis* OS261

**FIGURE 4:**
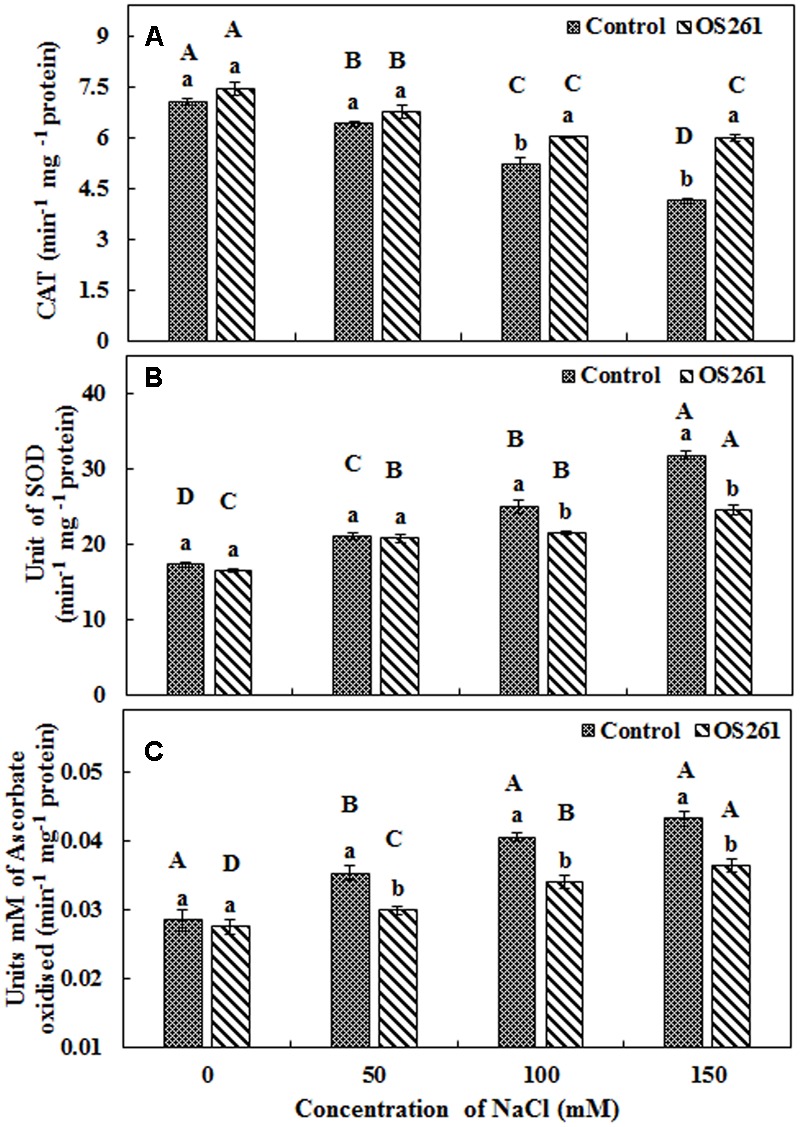
**Antioxidant enzymatic activities (A)** Catalase, **(B)** Superoxide dismutase, **(C)** Ascorbate peroxidase. Three replicates were used for each treatment. Different letters indicate significant differences *P* < 0.05 among the treatments at each salt levels (a, b) or among salt levels for each treatment: Control (A, B, C, D), OS261 (A, B, C, D). Each value represents the mean of three replicates ± standard error (SE).

**FIGURE 5:**
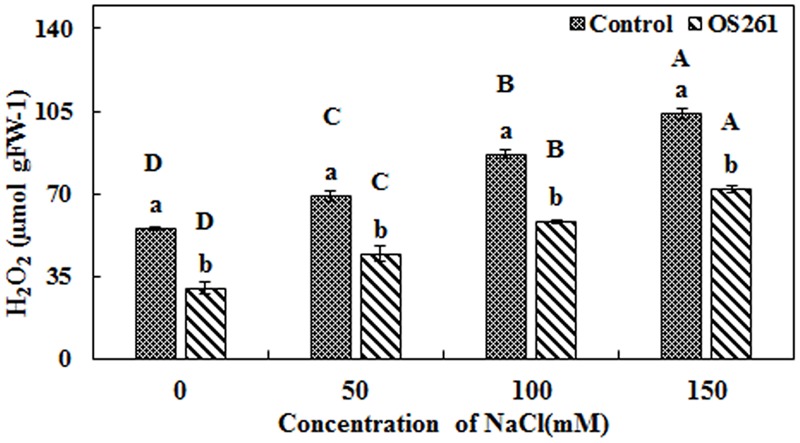
**Effect of *P. frederiksbergensis* OS261 inoculation on hydrogen peroxide content in red pepper plants under salinity stress.** Different letters indicate significant differences *P* < 0.05 among the treatments at each salt levels (a, b) or among salt levels for each treatment: Control (A, B, C, D), OS261 (A, B, C, D). Each value represents the mean of three replicates ± standard error (SE).

The activity of three antioxidant enzymes (CAT, SOD, and APX) varied under salt stress condition compared to the normal conditions. The CAT activity in the plants seemed to increase significantly after the treatment with the bacterium under the different levels of salt concentrations (**Figure 4A**). The inoculated plants showed 5.37, 5.62, 15.96, and 45.45% increase in CAT activity at 0, 50, 100, and 150 mM of the salt treatments. In the inoculated plants, the SOD activity showed no significant changes upon the inoculation at 0 and 50 mM salt concentration but was significantly reduced at 100 and 150 mM salt concentrations in comparison to the un-inoculated control plants (**Figure 4B**). Similarly, APX activity (**Figure 4C**) showed a similar pattern with SOD where bacterial inoculation significantly reduced the activity under 50, 100, and 150 mM of salt concentrations with no significant changes at 0 mM compared to the un-inoculated plants. Hydrogen peroxide content increased considerably in the un-inoculated control plants with an increase in the soil salinity (**Figure 5**). Interestingly, the bacterial inoculation reduced the peroxide content significantly, which may help the plant cope up with the effect of stress.

## Discussion

The use of ACC deaminase producing PGPR in soil resulted in an enhanced growth of red pepper plants under salt stress. These positive effects were exerted by maintaining the low levels of ethylene and modulating the expression of ROS-scavenging enzymes. Ethylene is a plant growth regulator, which is involved in several physiological responses (Abeles et al., 1992). In addition to that, it is also a stress hormone as it is synthesized rapidly under stress (Stearns and Glick, 2003). Several studies (Belimov et al., 2001; Saravanakumar and Samiyappan, 2007) have reported that ethylene reduces seed germination rate and root development during stress, which eventually affects plant growth. Microorganisms synthesizing the ACC deaminase enzyme can cleave ACC to α-ketobutyrate and ammonia, thereby decreasing ethylene stress in plants (Glick, 2005; Sun et al., 2009; Siddikee et al., 2011; Rashid et al., 2012). In this study, a PGPR strain *P*. *frederiksbergensis* OS261 which possesses ACC deaminase activity demonstrated its effectiveness in inducing salt tolerance and consequent improvement in the growth of pepper plants under salt stress.

Pepper (*Capsicum* spp.) along with tomato and potato is an economically important genus of Solanaceae family. Owing to its ability to adapt to different climates and being available in varieties of shapes, sizes and color, pepper plants are cultivated all over the world (Qin et al., 2014). Various biotic stresses such as viruses and abiotic stresses especially drought and salinity are known to affect this economically important family. Aktas et al. (2006) reported that pepper plants are highly susceptible to salinity stress. In plant growth experiments, pepper plants exhibited a decrease in plant height with an increase in the salinity in the un-inoculated controls. Salinity stress is one of the main obstacles that limit higher crop production. It increases the osmotic potential of the growth medium resulting the seeds to require more energy for water absorption, which leads to poor germination (Jamil and Rha, 2004). It decreases the rate of photosynthesis by restricting the photosynthetic electron transport, which in turn paralyzes the whole plant mechanism for survival and development (Hameed et al., 2015). In the present study, in contrast to the un-inoculated plants, the inoculated or bacterized plants showed enhancement in the growth despite increasing salinity. It indicates the ACC deaminase producing isolate *P*. *frederiksbergensis* OS261 is able to ameliorate the effect of salt on growth medium. Our results were consistent with the other findings (Mayak et al., 2004; Madhaiyan et al., 2006; Zahir et al., 2009; Ahmad et al., 2012; Cheng et al., 2012), where ACC deaminase-producing bacteria enhanced the growth of red pepper plants compared to un-inoculated control. Additionally, the stimulation of different crops by PGPR has been verified in both laboratory and field trials. *P. putida* and *P. fluorescens* have been found to play a considerable role in root and shoot elongation in canola, lettuce, and tomato (Kloepper et al., 1988; Hall et al., 1996) as well as crop yields in potato, radish, rice, sugar beet, tomato, lettuce, apple, citrus, beans, some ornamental plants, and wheat (Kloepper et al., 1988; Lemanceau, 1992; Kloepper, 1994). Interestingly, a study by Li et al. (2000) and Madhaiyan et al. (2006) observed that PGPR with ACC deaminase mutants could not modulate plant growth whereas ACC deaminase transgenic canola plants showed improved growth under salinity stress (Sergeeva et al., 2006). On the basis of the earlier studies, it can be suggested that ACC deaminase producing bacteria can promote plant growth and increase dry mass under salinity stress by cleaving ACC, which consequently reduces ethylene levels.

Abiotic stress such as salinity leads to ROS formation especially superoxide, hydroxyl ion, singlet oxygen and hydrogen peroxide, which results in the oxidation of cell membranes thereby causing damage to membrane and cell structures. These processes are called as oxidative stress (Apel and Hirt, 2004; Hussain et al., 2013). In plants, a defensive system consisting of ROS scavenging enzymes namely SOD, peroxidase (POD), glutathione reductase (GR), mono-hydroascorbate reductase (MDHAR), APX, CAT and redox ions work in a concerted way to carry out ROS detoxification (Mittler, 2002; Abogadallah, 2011; Abbas et al., 2013).

The pepper plants inoculated with ACC deaminase-containing PGPR exhibited a significant elevation of the antioxidant enzyme CAT compared to the un-inoculated plants suggesting that they were adapted to saline conditions by eliminating ROS. Our results were consistent with Gururani et al. (2013), where the CAT activity was enhanced in the inoculated potato plants under salt stress and also Subramanian et al. (2015), where the enzyme activity was improved in the inoculated tomato plants under cold stress. In the bacterium inoculated plants, the SOD activity showed a significant decrease in higher salinity levels suggesting neutralization of potentially harmful molecules leading to alleviation of salt stress (Cavalcanti et al., 2004). Similarly, the APX activity showed a pattern similar to SOD, where the bacterial inoculation significantly reduced the enzyme activity under salt stress (Han and Lee, 2005; Upadhyay et al., 2012). The reason behind the decrease may be the lack of hydrogen peroxide production in the plants inoculated with bacteria, where PGPR played a major role in maintaining the normal homeostasis of plant system (Gururani et al., 2013). The antioxidant enzyme activities have been studied extensively, but still the significance of enzymes in salt tolerance is not clear, as high antioxidant activities are linked to both salt tolerance and sensitivity (Abogadallah, 2010). Many studies (Shalata and Tal, 1998; Shalata et al., 2001; Mittova et al., 2003; Sumithra et al., 2006; Hediye Sekmen et al., 2007) have related the salt tolerance to an increase in the activity of antioxidant enzymes, whereas on the contrary, few studies (Cavalcanti et al., 2004; Kim et al., 2004) have shown that a higher salt tolerance does not depend always on a higher antioxidant activity.

## Conclusion

The present study illustrated that *P*. *frederiksbergensis* OS261 can promote salt stress tolerance in red pepper by regulating ethylene biosynthesis and increasing CAT enzyme activity. A further investigation on the genes responsible for CAT enzyme may reveal the exact mechanism by which *P*. *frederiksbergensis* OS261 enhances salt stress tolerance. This strain has already been well studied and proved to enhance plant cold stress tolerance. The use of this strain as a bio-inoculant can also enhance plant growth under other stress conditions. Therefore, it may be recommended to the farmer as a stress mitigator for the regions with high soil salinity after assessing its field level performance.

## Author Contributions

PC, SS, and TS: conception and design of the work. PC, SS, and YK: performed the work. PC, SS, YK and KK: acquisition of data. PC, SS, GS and KK: analyzed the data. PC, SS, GS, RA and TS: critical revision of manuscript. PC, SS, RA and TS: wrote the paper.

## Conflict of Interest Statement

The authors declare that the research was conducted in the absence of any commercial or financial relationships that could be construed as a potential conflict of interest.

## References

[B1] Abbas T. Pervez M. A. Ayyub C. M. Ahmad R. (2013). Assessment of morphological, antioxidant, biochemical and ionic responses of salt-tolerant and salt-sensitive okra (*Abelmoschus esculentus*) under saline regime. *Pakistan J. Life Soc. Sci.* 11 147–153.

[B2] Abeles F. B. Morgan P. W. Saltveit M. E. Jr. (1992). *Ethylene in Plant Biology* 2nd Edn. San Diego, CA: Academic Press Inc.

[B3] Abogadallah G. M. (2010). Insights into the significance of antioxidative defense under salt stress. *Plant Signal. Behav.* 5 369–374. 10.4161/psb.5.4.10873 20118663 PMC2958586

[B4] Abogadallah G. M. (2011). Differential regulation of photorespiratory gene expression by moderate and severe salt and drought stress in relation to oxidative stress. *Plant Sci.* 180 540–547. 10.1016/j.plantsci.2010.12.004 21421402

[B5] Ahmad M. Zahir Z. A. Asghar H. N. Arshad M. (2012). The combined application of rhizobial strains and plant growth promoting rhizobacteria improves growth and productivity of mung bean (*Vigna radiata* L.) under salt-stressed conditions. *Ann. Microbiol.* 62 1321–1330. 10.1007/s13213-011-0380-9

[B6] Aktas H. Abak K. Cakmak I. (2006). Genotypic variation in the response of pepper to salinity. *Sci. Hortic.* 110 260–266. 10.1016/j.scienta.2006.07.017

[B7] Allakhverdiev S. I. Sakamoto A. Nishiyama Y. Inaba M. Murata N. (2000). Ionic and osmotic effects of NaCl-induced inactivation of photosystems I and II in *Synechococcus* sp. *Plant Physiol.* 123 1047–1056. 10.1104/pp.123.3.1047 10889254 PMC59068

[B8] Apel K. Hirt H. (2004). Reactive oxygen species: metabolism, oxidative stress, and signal transduction. *Annu. Rev. Plant Biol.* 55 373–399. 10.1146/annurev.arplant.55.031903.141701 15377225

[B9] Bacilio M. Moreno M. Bashan Y. (2016). Mitigation of negative effects of progressive soil salinity gradients by application of humic acids and inoculation with *Pseudomonas stutzeri* in a salt-tolerant and a salt-susceptible pepper. *Appl. Soil Ecol.* 107 394–404. 10.1016/j.apsoil.2016.04.012

[B10] Bano A. Fatima M. (2009). Salt tolerance in *Zea mays* (L). following inoculation with *Rhizobium* and *Pseudomonas*. *Biol. Fertil. Soils* 45 405–413. 10.1007/s00374-008-0344-9

[B11] Belimov A. A. Safronova V. I. Sergeyeva T. A. Egorova T. N. Matveyeva V. A. Tsyganov V. E. (2001). Characterization of plant growth promoting rhizobacteria isolated from polluted soils and containing 1-aminocyclopropane-1-carboxylate deaminase. *Can. J. Microbiol.* 47 642–652. 10.1139/w01-062 11547884

[B12] Bharti N. Yadav D. Barnawal D. Maji D. Kalra A. (2013). *Exiguobacterium oxidotolerans*, a halotolerant plant growth promoting rhizobacteria, improves yield and content of secondary metabolites in *Bacopa monnieri* (L.) Pennell under primary and secondary salt stress. *World J. Microbiol. Biotechnol.* 29 379–387. 10.1007/s11274-012-1192-1 23085953

[B13] Bojórquez-Quintal E. Velarde-Buendía A. Ku-González A. Carillo-Pech M. Ortega-Camacho D. Echevarría-Machado I. (2014). Mechanisms of salt tolerance in habanero pepper plants (*Capsicum chinense* Jacq.): proline accumulation, ions dynamics and sodium root-shoot partition and compartmentation. *Front. Plant Sci.* 5:605 10.3389/fpls.2014.00605 PMC4228851 25429292

[B14] Cavalcanti F. R. Oliveira J. T. A. Martins-Miranda A. S. Viégas R. A. Silveira J. A. G. (2004). Superoxide dismutase, catalase and peroxidase activities do not confer protection against oxidative damage in salt-stressed cowpea leaves. *New Phytol.* 163 563–571. 10.1111/j.1469-8137.2004.01139.x 33873746

[B15] Cheng Z. Park E. Glick B. R. (2007). 1-Aminocyclopropane-1-carboxylate deaminase from *Pseudomonas putida* UW4 facilitates the growth of canola in the presence of salt. *Can. J. Microbiol.* 53 912–918. 10.1139/W07-050 17898846

[B16] Cheng Z. Woody O. Z. McConkey B. J. Glick B. R. (2012). Combined effects of the plant growth-promoting bacterium *Pseudomonas putida* UW4 and salinity stress on the *Brassica napus* proteome. *Appl. Soil Ecol.* 61 255–263. 10.1016/j.apsoil.2011.10.006

[B17] Chojak-Koźniewska J. Linkiewicz A. Sowa S. Radzioch M. A. Kuźniak E. (2017). Interactive effects of salt stress and *Pseudomonas syringae* pv. lachrymans infection in cucumber: involvement of antioxidant enzymes, abscisic acid and salicylic acid. *Environ. Exp. Bot.* 136 9–20. 10.1016/j.envexpbot.2017.01.004

[B18] Ding S. Huang C. L. Sheng H. M. Song C. L. Li Y. B. An L. Z. (2011). Effect of inoculation with the endophyte *Clavibacter* sp. strain Enf12 on chilling tolerance in *Chorispora bungeana*. *Physiol. Plant.* 141 141–151. 10.1111/j.1399-3054.2010.01428.x 21044086

[B19] Dodd I. C. Pérez-Alfocea F. (2012). Microbial amelioration of crop salinity stress. *J. Exp. Bot.* 63 3415–3428. 10.1093/jxb/ers033 22403432

[B20] Forieri I. Hildebrandt U. Rostás M. (2016). Salinity stress effects on direct and indirect defence metabolites in maize. *Environ. Exp. Bot.* 122 68–77. 10.1016/j.envexpbot.2015.09.007

[B21] Glick B. Penrose D. Li J. (1998). A model for the lowering of plant ethylene concentrations by plant growth-promoting bacteria. *J. Theor. Biol.* 190 63–68. 10.1006/jtbi.1997.0532 9473391

[B22] Glick B. R. (2005). Modulation of plant ethylene levels by the bacterial enzyme ACC deaminase. *FEMS Microbiol. Lett.* 251 1–7. 10.1016/j.femsle.2005.07.030 16099604

[B23] Goth L. (1991). A simple method for determination of serum catalase activity and revision of reference range. *Clin. Chim. Acta* 196 143–151. 10.1016/0009-8981(91)90067-M 2029780

[B24] Gururani M. A. Upadhyaya C. P. Baskar V. Venkatesh J. Nookaraju A. Park S. W. (2013). Plant growth-promoting rhizobacteria enhance abiotic stress tolerance in *Solanum tuberosum* through inducing changes in the expression of ROS-scavenging enzymes and improved photosynthetic performance. *J. Plant Growth Regul.* 32 245–258. 10.1007/s00344-012-9292-6

[B25] Habib S. H. Kausar H. Saud H. M. (2016). Plant growth-promoting rhizobacteria enhance salinity stress tolerance in Okra through ROS-scavenging enzymes. *Biomed. Res. Int.* 2016:6284547 10.1155/2016/6284547 PMC4756578 26951880

[B26] Hall J. A. Peirson D. Ghosh S. Glick B. R. (1996). Root elongation in various agronomic crops by the plant growth promoting rhizobacterium *Pseudomonas putida* GR12–2. *Isr. J. Plant Sci.* 44 37–42. 10.1080/07929978.1996.10676631

[B27] Hamdia M. A. Shaddad M. A. K. Doaa M. M. (2004). Mechanisms of salt tolerance and interactive effects of *Azospirillum brasilense* inoculation on maize cultivars grown under salt stress conditions. *Plant Growth Regul.* 44 165–174. 10.1007/s10725-004-3131-0

[B28] Hameed A. Gulzar S. Aziz I. Hussain T. Gul B. Khan M. A. (2015). Effects of salinity and ascorbic acid on growth, water status and antioxidant system in a perennial halophyte. *AoB Plants* 7:lv004 10.1093/aobpla/plv004 PMC4334656 25603966

[B29] Han H. S. Lee K. D. (2005). Plant growth promoting rhizobacteria effect on antioxidant status, photosynthesis, mineral uptake and growth of lettuce under soil salinity. *Res. J. Agric. Biol. Sci.* 1 210–215.

[B30] Hediye Sekmen A. Türkan I. Takio S. (2007). Differential responses of antioxidative enzymes and lipid peroxidation to salt stress in salt-tolerant *Plantago maritima* and salt-sensitive *Plantago media*. *Physiol. Plant.* 131 399–411. 10.1111/j.1399-3054.2007.00970.x 18251879

[B31] Hussain S. Khaliq A. Matloob A. Wahid M. A. Afzal I. (2013). Germination and growth response of three wheat cultivars to NaCl salinity. *Soil Environ.* 32 36–43.

[B32] Islam M. R. Sultana T. Joe M. M. Yim W. Cho J. C. Sa T. (2013). Nitrogen-fixing bacteria with multiple plant growth-promoting activities enhance growth of tomato and red pepper. *J. Basic Microbiol.* 53 1004–1015. 10.1002/jobm.201200141 23553337

[B33] Jamil M. Rha E. S. (2004). The effect of salinity (NaCl) on the germination and seedling of sugar beet (*Beta vulgaris* L.) and cabbage (*Brassica oleracea* L.). *Plant Resour.* 7 226–232.

[B34] Kim Y. Arihara J. Nakayama T. Nakayama N. Shimada S. Usui K. (2004). Antioxidative responses and their relation to salt tolerance in *Echinochloa oryzicola* vasing and *Setaria virdis* (L.) Beauv. *Plant Growth Regul.* 44 87–92. 10.1007/s10725-004-2746-5

[B35] Kloepper J. W. (1994). “Plant growth-promoting rhizobacteria (other systems),” in *Azospirillum/Plant Associations* ed. Okon Y. (Boca Raton, FL: CRC Press) 137–166.

[B36] Kloepper J. W. Lifshitz R. Schroth M. N. (1988). *Pseudomonas* inoculants to benefit plant production. *ISI Atlas Sci. Anim. Plant Sci.* 1 60–64. 10.1007/s00253-009-2092-7

[B37] Lemanceau P. (1992). Effects bénefiqués de rhizobactéries sur les plants: example des *Pseudomonas* spp. *fluorescents*. *Agronomie* 12 413–437. 10.1051/agro:19920601

[B38] Li J. Ovakim D. H. Charles T. C. Glick B. R. (2000). An ACC deaminase minus mutant of *Enterobacter cloacae* UW4 no longer promotes root elongation. *Curr. Microbiol.* 41 101–105. 10.1007/s002840010101 10856374

[B39] Madhaiyan M. Poonguzhali S. Ryu J. Sa T. (2006). Regulation of ethylene levels in canola (*Brassica campestris*) by 1-aminocyclopropane-1-carboxylate deaminase-containing *Methylobacterium fujisawaense*. *Planta* 224 268–278. 10.1007/s00425-005-0211-y 16416316

[B40] Mayak S. Tirosh T. Glick B. R. (2004). Plant growth-promoting bacteria confer resistance in tomato plants to salt stress. *Plant Physiol. Biochem.* 42 565–572. 10.1016/j.plaphy.2004.05.009 15246071

[B41] Mittler R. (2002). Oxidative stress, antioxidants and stress tolerance. *Trends Plant Sci.* 7 405–410. 10.1016/S1360-1385(02)02312-9 12234732

[B42] Mittova V. Tal M. Volokita M. Guy M. (2003). Up-regulation of the leaf mitochondrial and peroxisomal antioxidative systems in response to salt-induced oxidative stress in the wild salt-tolerant tomato species *Lycopersicon pennellii*. *Plant Cell Environ.* 26 845–856. 10.1046/j.1365-3040.2003.01016.x 12803612

[B43] Morgan P. W. Drew M. C. (1997). Ethylene and plant responses to stress. *Physiol. Plant.* 100 620–630. 10.1034/j.1399-3054.1997.1000325.x

[B44] Munne-Bosch S. Pinto-Marijuan M. (2016). Free radicals, oxidative stress and antioxidants. *Encycl. Appl. Plant Sci.* 2 16–19. 10.1016/b978-0-12-394807-6.00077-0

[B45] Munns R. Tester M. (2008). Mechanisms of salinity tolerance. *Annu. Rev. Plant Biol.* 59 651–681. 10.1146/annurev.arplant.59.032607.092911 18444910

[B46] Patra J. K. Das G. Paramithiotis S. Shin H. S. (2016). Kimchi and other widely consumed traditional fermented foods of Korea: a review. *Front. Microbiol.* 7:1493 10.3389/fmicb.2016.01493 PMC5039233 27733844

[B47] Qin C. Yu C. Shen Y. Fang X. Chen L. Min J. (2014). Whole-genome sequencing of cultivated and wild peppers provides insights into *Capsicum* domestication and specialization. *Proc. Natl. Acad. Sci. U.S.A.* 111 5135–5140. 10.1073/pnas.1400975111 24591624 PMC3986200

[B48] Rashid S. Charles T. C. Glick B. R. (2012). Isolation and characterization of new plant growth-promoting bacterial endophytes. *Appl. Soil Ecol.* 61 217–224. 10.1016/j.apsoil.2011.09.011

[B49] Sandipan S. Parthiban S. Melvin Jeo M. Chanratana M. Poulami C. Sa T. (2016). “Plant growth promoting characteristics of *Pseudomonas frederiksbergensis* OS261 and its effect on early growth of red pepper plants under greenhouse conditions,” in *Proceedings of the Korea Society of Soil Science and Fertilizer Day 2016 Celebrates the Spring Conference of the Republic of Korea Soil* Seoul 61–62.

[B50] Sang M. K. Chun S. C. Kim K. D. (2008). Biological control of *Phytophthora* blight of pepper by antagonistic rhizobacteria selected from a sequential screening procedure. *Biol. Control* 46 424–433. 10.1016/j.biocontrol.2008.03.017

[B51] Saravanakumar D. Samiyappan R. (2007). ACC deaminase from *Pseudomonas fluorescens* mediated saline resistance in groundnut (*Arachis hypogea*) plants. *J. Appl. Microbiol.* 102 1283–1292. 10.1111/j.1365-2672.2006.03179.x 17448163

[B52] Schubert S. Neubert A. Schierholt A. Sümer A. Zörb C. (2009). Development of salt-resistant maize hybrids: the combination of physiological strategies using conventional breeding methods. *Plant Sci.* 177 196–202. 10.1016/j.plantsci.2009.05.011

[B53] Sergeeva E. Shah S. Glick B. R. (2006). Growth of transgenic canola (*Brassica napus* cv. Westar) expressing a bacterial 1-aminocyclopropane-1-carboxylate (ACC) deaminase gene on high concentrations of salt. *World J. Microbiol. Biotechnol.* 22 277–282. 10.1007/s11274-005-9032-1

[B54] Shalata A. Mittova V. Volokita M. Guy M. Tal M. (2001). Response of the cultivated tomato and its wild salt-tolerant relative *Lycopersicon pennellii* to salt-dependent oxidative stress: the root antioxidative system. *Physiol. Plant.* 112 487–494. 10.1034/j.1399-3054.2001.1120405.x 11473708

[B55] Shalata A. Tal M. (1998). The effect of salt stress on lipid peroxidation and antioxidants in the leaf of the cultivated tomato and its wild salt-tolerant relative *Lycopersicon pennellii*. *Physiol. Plant.* 104 169–174. 10.1034/j.1399-3054.1998.1040204.x 11473708

[B56] Siddikee M. A. Chauhan P. S. Anandham R. Han G. H. Sa T. (2010). Isolation, characterization, and use for plant growth promotion under salt stress, of ACC deaminase-producing halotolerant bacteria derived from coastal soil. *J. Microbiol. Biotechnol.* 20 1577–1584. 10.4014/jmb.1007.07011 21124065

[B57] Siddikee M. A. Glick B. R. Chauhan P. S. Yim W. J. Sa T. (2011). Enhancement of growth and salt tolerance of red pepper seedlings (*Capsicum annuum* L.) by regulating stress ethylene synthesis with halotolerant bacteria containing 1-aminocyclopropane-1-carboxylic acid deaminase activity. *Plant Physiol. Biochem.* 49 427–434. 10.1016/j.plaphy.2011.01.015 21300550

[B58] Siddikee M. A. Sundaram S. Chandrasekaran M. Kim K. Selvakumar G. Sa T. (2015). Halotolerant bacteria with ACC deaminase activity alleviate salt stress effect in canola seed germination. *J. Korean Soc. Appl. Biol. Chem.* 58 237–241. 10.1007/s13765-015-0025-y

[B59] Singh R. P. Jha P. N. (2016). Alleviation of salinity-induced damage on wheat plant by an ACC deaminase-producing halophilic bacterium *Serratia* sp. SL- 12 isolated from a salt lake. *Symbiosis* 69 101–111. 10.1007/s13199-016-0387-x

[B60] Stavridou E. Hastings A. Webster R. J. Robson P. R. H. (2016). The impact of soil salinity on the yield, composition and physiology of the bioenergy grass *Miscanthus* × *giganteus*. *GCB Bioenergy* 9 92–104. 10.1111/gcbb.12351

[B61] Stearns J. C. Glick B. R. (2003). Transgenic plants with altered ethylene biosynthesis or perception. *Biotechnol. Adv.* 21 193–210. 10.1016/S0734-9750(03)00024-7 14499129

[B62] Subramanian P. Kim K. Krishnamoorthy R. Mageswari A. Selvakumar G. Sa T. (2016). Cold stress tolerance in psychrotolerant soil bacteria and their conferred chilling resistance in tomato (*Solanum lycopersicum* Mill.) under low temperatures. *PLoS ONE* 11:e0161592 10.1371/journal.pone.0161592 PMC5006972 27580055

[B63] Subramanian P. Mageswari A. Kim K. Lee Y. Sa T. (2015). Psychrotolerant endophytic *Pseudomonas* sp. strains OB155 and OS261 induced chilling resistance in tomato plants (*Solanum lycopersicum* Mill.) by activation of their antioxidant capacity. *Mol. Plant Microbe Interact.* 28 1073–1081. 10.1094/MPMI-01-15-0021-R 26075827

[B64] Sumithra K. Jutur P. P. Carmel B. D. Reddy A. R. (2006). Salinity-induced changes in two cultivars of *Vigna radiata*: responses of antioxidative and proline metabolism. *Plant Growth Regul.* 50 11–22. 10.1007/s10725-006-9121-7

[B65] Sun Y. Cheng Z. Glick B. R. (2009). The presence of a1-aminocyclopropane-1-carboxylate (ACC) deaminase deletion mutation alters the physiology of the endophytic plant growth-promoting bacterium *Burkholderia phytofirmans* PsJN. *FEMS Microbiol. Lett.* 296 131–136. 10.1111/j.1574-6968.2009.01625.x 19459964

[B66] Tank N. Saraf M. (2010). Salinity-resistant plant growth promoting rhizobacteria ameliorates sodium chloride stress on tomato plants. *J. Plant Interact.* 5 51–58. 10.1080/17429140903125848

[B67] Theocharis A. Bordiec S. Fernandez O. Paquis S. Dhondt-Cordelier S. Baillieul F. (2012). *Burkholderia phytofirmans* PsJN primes *Vitis vinifera* L. and confers a better tolerance to low nonfreezing temperatures. *Mol. Plant Microbe Interact.* 25 241–249. 10.1094/MPMI-05-11-0124 21942451

[B68] Upadhyay S. K. Singh J. S. Saxena A. K. Singh D. P. (2012). Impact of PGPR inoculation on growth and antioxidant status of wheat under saline conditions. *Plant Biol.* 14 605–611. 10.1111/j.1438-8677.2011.00533.x 22136617

[B69] Yaish M. W. Al-Lawati A. Jana G. A. Patankar H. V. Glick B. R. (2016). Impact of soil salinity on the structure of the bacterial endophytic community identified from the roots of caliph medic (*Medicago truncatula*). *PLoS ONE* 11:e0159007 10.1371/journal.pone.0159007 PMC4938511 27391592

[B70] Yang J. Kloepper J. W. Ryu C. M. (2009). Rhizosphere bacteria help plants tolerate abiotic stress. *Trends Plant Sci.* 14 1–4. 10.1016/j.tplants.2008.10.004 19056309

[B71] Zahir Z. A. Ghani U. Naveed M. Nadeem S. M. Asghar H. N. (2009). Comparative effectiveness of *Pseudomonas* and *Serratia* sp. containing ACC-deaminase for improving growth and yield of wheat (*Triticum aestivum* L.) under salt-stressed conditions. *Arch. Microbiol.* 191 415–424. 10.1007/s00203-009-0466-y 19255743

